# Biodegradable Chitosan Nanoparticle Coatings on Titanium for the Delivery of BMP-2

**DOI:** 10.3390/biom5010003

**Published:** 2015-01-08

**Authors:** Nils Poth, Virginia Seiffart, Gerhard Gross, Henning Menzel, Wibke Dempwolf

**Affiliations:** 1 Institut für Technische Chemie, Technische Universität Braunschweig, Hans-Sommer-Str. 10, 38106 Braunschweig, Germany; E-Mails: n.poth@tu-bs.de (N.P.); h.menzel@tu-bs.de (H.M.); 2 Helmholtz-Zentrum für Infektionsforschung, Signaling and Gene Regulation, 38124 Braunschweig, Germany; E-Mails: virginia.seiffart@uk-essen.de (V.S.); gerhard.gross@helmholtz-hzi.de (G.G.)

**Keywords:** BMP-2 delivery, nanoparticle degradation, coatings, chitosan, biodegradable

## Abstract

A simple method for the functionalization of a common implant material (Ti6Al4V) with biodegradable, drug loaded chitosan-tripolyphosphate (CS-TPP) nanoparticles is developed in order to enhance the osseointegration of endoprostheses after revision operations. The chitosan used has a tailored degree of acetylation which allows for a fast biodegradation by lysozyme. The degradability of chitosan is proven via viscometry. Characteristics and degradation of nanoparticles formed with TPP are analyzed using dynamic light scattering. The particle degradation via lysozyme displays a decrease in particle diameter of 40% after 4 days. Drug loading and release is investigated for the nanoparticles with bone morphogenetic protein 2 (BMP-2), using ELISA and the BRE luciferase test for quantification and bioactivity evaluation. Furthermore, nanoparticle coatings on titanium substrates are created via spray-coating and analyzed by ellipsometry, scanning electron microscopy and X-ray photoelectron spectroscopy. Drug loaded nanoparticle coatings with biologically active BMP-2 are obtained *in vitro* within this work. Additionally, an *in vivo* study in mice indicates the dose dependent induction of ectopic bone growth through CS-TPP-BMP-2 nanoparticles. These results show that biodegradable CS-TPP coatings can be utilized to present biologically active BMP-2 on common implant materials like Ti6Al4V.

## 1. Introduction

A big challenge in orthopedics still lies in the process of aseptic prosthesis loosening which is the reason for more than 80% of the carried out revision operations in the field of endoprostheses [1]. The aseptic loosening is mainly caused by “stress shielding” and osteolysis due to abrasion particles [1,2]. Induced bone remodeling and osteolysis lead to gap formation at the implant/bone interface followed by continuously decreasing osseointegration of the implant. This gives rise to inflammations, prosthesis loosening and revision operations. In Germany alone, about 37,000 revision operations were carried out in 2012 according to the “Federal Statistical Office Germany” [3]. In most revision cases, a loose implant has to be explanted and a damaged bone is left behind. Placing a new implant into the partially osteolyzed spongia is difficult compared to the first implantation due to less implant/bone contact. Right after the revision a “race for the surface” of bacteria and tissue cells takes place which, in case of bacteria winning the race, leads to inflammatory reactions interfering with the ingrowth of the implant [4]. A functionalized implant surface that supports tissue cells during the “race for the surface” in order to achieve an efficient and fast osseointegration of the implant would therefore be a major benefit for the affected patients.

A well-known drug for the enhancement of *in vivo* bone formation is the signaling protein bone morphogenetic protein 2 (BMP-2) [5,6]. The ultilization of BMP-2 as part of an implant coating is to promote osteoblasts proliferation, differentiation and attachment during the “race for the surface”. This effective support of osteoblasts might be a way to induce bone formation right in the gaps at the implant/bone interface, ideally resulting in a better osseointegration of the implant [7]. The osteoinductive effect of BMP-2 has already been described in a wide range of publications [6,8]. Concerning different BMP-2 delivery strategies, Li *et al.* provided a detailed overview [9]. Chatzinikolaidou *et al.* published data that emphasizes the ability of BMP-2 to enhance osseointegration of titanium substrates *in vivo* [10]. Moreover, the bone growth promoting effect of BMP‑2 was reported by Hayashi *et al.* In their work, enhanced osteoblast activity and induced bone growth could be shown using cholesterol modified pullulan nanogels for the transport of BMP-2 [11]. Mendoza-Palomares *et al.* demonstrated bone and cartilage regeneration via employing a biomimicking membrane with nanoreservoirs for BMP-2 [12]. Several other studies confirm that BMP-2 promotes osteoblast activity *in vitro* via increased alkaline phosphatase (ALP) activity and calcium mineral deposition [13,14,15,16]. *In vivo* studies have been performed as well: Abarrategi *et al.* investigated chitosan/rhBMP-2 films on titanium substrates *in vivo*. Their results show a noticeable formation of bone around chitosan/rhBMP-2 coated implants compared to the uncoated controls [16]. Additional *in vivo* investigations from Facca *et al.* and Shah *et al.* support the *in vivo* bone growth promoting activity of BMP-2 in a multilayered capsules and a multilayer coating system [17,18].

The way the signaling protein is immobilized at the implant surface plays an important role for its biological activity. A broad spectrum of different approaches for the immobilization of BMP‑2 can be found in literature. Important methods are the adsorption on chromo sulfuric acid treated titanium [19], the adsorption on silanized titanium [20], covalent immobilization with polymers [21], covalent immobilization via a copolymer [22], binding to self-assembled monolayers [23], the incorporation in polyelectrolyte multilayers [16,24] and the incorporation in hydrogel scaffolds [25] and nanocomplexes [26]. Since the adsorption and the covalent immobilization may result in a decrease of the bioactivity of the protein [23,27], our studies focus on the development of a well-adapted drug delivery system for the effective release of bioactive BMP-2 at the site of implantation.

It is stated in literature that chitosan (CS)-tripolyphosphate (TPP) nanoparticles are capable of incorporating and releasing a variety of drugs: Gan *et al.* showed the successful entrapment of bovine serum albumin (BSA) in CS-TPP nanoparticles followed by a diffusion based release of the protein [28]. Similar results were also reported by Li *et al.* for 5‑fluorouracil and leucovorin [29], by Ji *et al.* for gentamicin and salicylic acid [30], and by Alishahi *et al.* for vitamin C [31]. The incorporation of BMP-2 into CS-TPP nanoparticles has not been reported yet. Immobilizing BMP-2 loaded CS-TPP nanoparticles on a titanium surface is thought to lead to a controlled release of unmodified, biologically active BMP-2. This way BMP-2 is assumed to be delivered locally in sufficient amounts resulting in stimulated bone growth and allowing for a better osseointegration of functionalized implants. Besides the action of the protein, the CS-TPP system is supposed to provide additional support due to osteoinductive properties of chitosan stated by Leedy *et al.* [32]. In detail, the degradation products of chitosan (chitosan oligomers) are to promote BMP-2 and ALP production [33,34]. ALP is a marker for the differentiation of osteoblast-like cells and a keyplayer in early bone mineralization [35]. Furthermore, ALP is needed for the degradation of TPP into monophosphate units, which are essential for the creation of bone [36].

All in all the approach of loading BMP-2 into CS-TPP nanoparticles adsorbed to the implant surface is thought to yield a simple alternative for the delivery of BMP-2. Presumably, a local release of biologically active BMP-2 can be achieved which, in cooperation with the beneficial effects of the drug carrying material, leads to enhanced osseointegration properties of functionalized implants.

## 2. Results and Discussion

### 2.1. Chitosan Purification and Acetylation

For the safe use of chitosan in medical applications and as a drug delivery system in the human organism chitosan has to be purified due to impurities like proteins. The purified chitosan is residue-free soluble and yields a clear, colorless solution as described by Gan *et al.* [37].

The following acetylation of chitosan was carried out because of preliminary experiments with CS with a degree of acetylation (DA) of 17%. These studies indicated that nearly all the BMP-2 was incorporated in the CS(17)-TPP nanoparticles and ELISA release studies with BMP-2 loaded CS(17)-TPP nanoparticles revealed no release of BMP-2 into the supernatant via diffusion. Thus, it was concluded that BMP-2 is stuck in the particles and CS-TPP nanoparticles have to be biodegradable in order to achieve a release of the incorporated BMP-2. Biodegradability of chitosan, the main component of the prepared CS-TPP nanoparticles, largely depends on its DA and according to Freier *et al.* is mainly driven by the enzyme lysozyme [38]. Several literature reports indicate that the degradation rate of chitosan with a DA below 30% is very low, which would not be suitable for a fast release of BMP-2 from the nanoparticles. However, for chitosan with a DA around 40% a much faster degradation is reported [38,39]. Therefore, CS was acetylated resulting in an experimental DA of 42%, as determined via ^1^H-NMR. The acetylated chitosan (CS(42)) matched the estimated DA values given by Freier *et al.* [38] and is soluble in distilled water. Since the solubility of chitosan can be attributed to the number of glucosamine units in the polymer available for protonation, a decreasing number of free amino groups along with a better solubility may seem contradictory. This phenomenon has already been reported several times in literature but is not yet fully understood [38,40,41].

### 2.2. Chitosan Degradation: Viscometry

An elegant approach to mimic the biodegradation of chitosan lies in the utilization of enzymes which are already available in the human body. The enzyme lysozyme was chosen for the degradation of chitosan *in vitro*, since it is reported to be efficient in degrading a variety of different polysaccharides including chitosan [38,39]. Moreover, lysozyme can be found in various human body fluids [42,43]. According to Brouwer *et al.* and Venge *et al.* a lysozyme concentration of 1.5 µg/mL equals the concentration of lysozyme in human serum [42,44]. Taking into account that human serum is part of the human blood and bone is a well blood supplied tissue, lysozyme should be present at the site of implantation after an operation. Therefore, we found lysozyme to be a promising candidate for degradation studies on chitosan and chitosan based nanoparticles.

**Figure 1:**
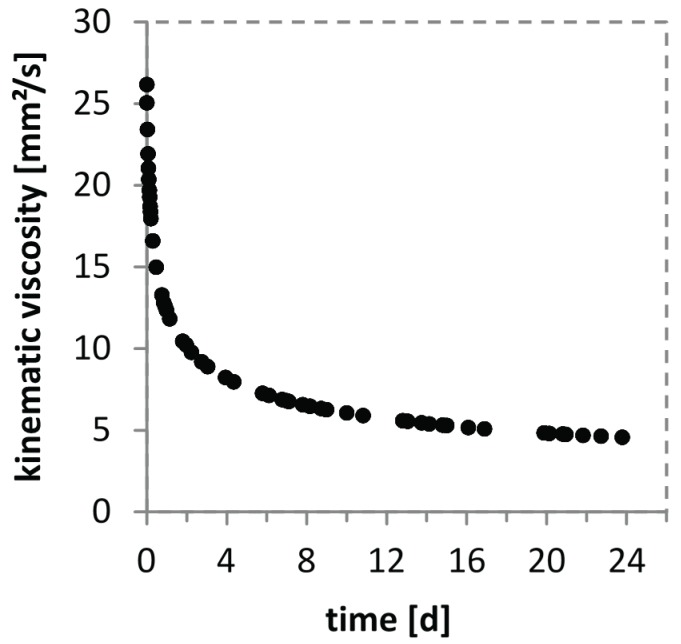
Time dependent, viscometrical tracking of the degradation of a 10 mg/mL chitosan solution (0.1% AcOH, DA 42%) with 1.5 µg/mL lysozyme at 37 °C.

In order to validate the degradability of CS(42) viscosity measurements were performed. For this purpose the kinematic viscosity of a chitosan solution at 37 °C with 1.5 µg/mL lysozyme was measured. As shown in Figure 1, it is obvious that lysozyme is able to degrade CS(42) even in light acidic media (0.1% AcOH). The degradation process was tracked for 24 days revealing a fast degradation during the first 4 days followed by a steadily down slowing of the degradation rate. The starting value for the kinematic viscosity of the solution is ~26 mm^2^/s going down to ~4.5 mm^2^/s after 24 days, after 4 days the kinematic viscosity already reached ~8 mm^2^/s. These results prove the ongoing degradation of CS(42) in the presence of lysozyme via a decrease in solution viscosity which is in agreement with degradation studies of Vårun *et al.* in human serum [45].

### 2.3. Nanoparticle Synthesis

There are several, slightly different methods for the preparation of CS-TPP nanoparticles reported in the literature [37,46]. The basic principle is to add TPP to an acetic acid/chitosan solution which spontaneously induces the formation of nanoparticles via the ionic gelation process depending on the concentration of TPP and chitosan solutions [47]. CS(42)-TPP (3:1, 1 mg/mL, 0.075% AcOH) nanoparticles created in this work possessed a particle size of 148 ± 72 nm with a polydispersity index (PDI) of 0.23 and a zeta potential of 23 ± 5 mV. The zeta potential of nanoparticles provides information about the surface charge of the analyzed nanoparticles, thus giving an idea of the strength of interaction between nanoparticles and the titanium surface as well as the stability of the nanoparticles. Raising the CS(42)-TPP ratio above 3:1 leads to broader particle distributions with increased particle sizes and zeta potentials, which is in agreement with other publications [48]. All experiments covered in this article were conducted using 3:1 CS(42)-TPP particles due to the easier characterization of narrowly distributed, nearly monomodal particles, even though a higher CS to TPP ratio would presumably promote the nanoparticle adsorption on titanium substrates on account of the higher positive zeta potential.

### 2.4. Nanoparticle Degradation

Degradable CS-TPP nanoparticles are essential for the release of the incorporated BMP-2 due to the absence of a diffusion based release of the protein according to our previously conducted ELISA release studies with CS(17)-TPP nanoparticles (data not shown here). The degradation behavior of chitosan itself in the presence of lysozyme with a concentration of 1.5 µg/mL has been shown in Figure 1 and has already been reported in several other studies for varying media, lysozyme concentrations and acetylation degrees [38,45,49,50]. In regard to the degradation of CS-TPP nanoparticles there are no enzyme based degradation studies in literature yet, though the incorporation of lysozyme into CS-TPP nanoparticles is investigated in some studies [51,52].

**Figure 2:**
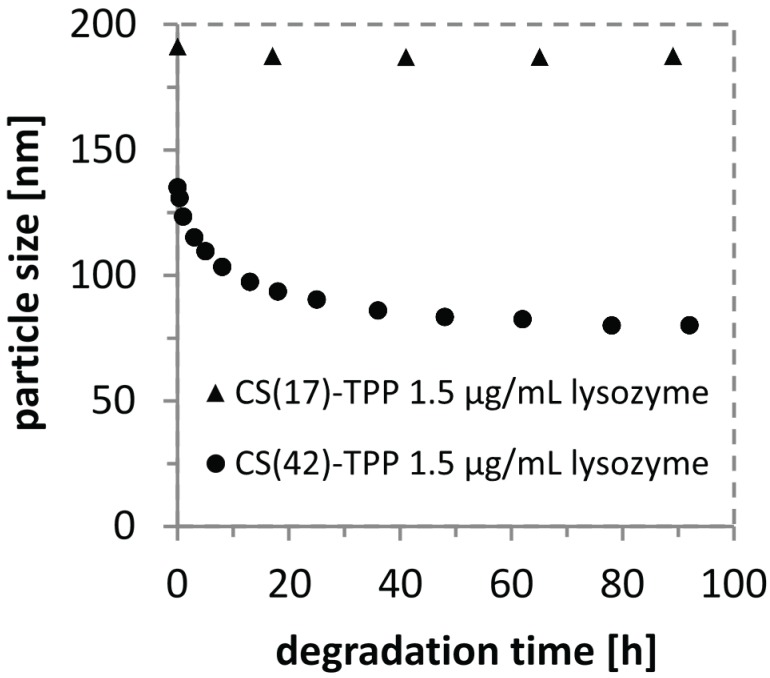
Plot of the particle size over the time during the degradation with 1.5 µg/mL lysozyme at 37 °C for chitosan-tripolyphosphate (3:1, 1 mg/mL, 0.075% AcOH, DA 17%) and (3:1, 1 mg/mL, 0.075% AcOH, DA 42%) nanoparticle solutions.

*In vitro* degradation experiments of CS(17)-TPP nanoparticles (3:1, 1 mg/mL, 0.075% AcOH) showed no particle degradation with lysozyme after one week. In contrast, CS(42)-TPP nanoparticles (3:1, 1 mg/mL, 0.075% AcOH) are degraded and show a decrease in particles size of ≈40% within 4 days as shown in Figure 2. A fast decrease in particle size can be observed in the first 10 h followed by a moderate to slow decrease phase up to 7 days. Beyond a week of degradation no further particle size changes could be detected. Loading lysozyme into CS-TPP nanoparticles has already been performed by Deng *et al.* and Piras *et al.*, but only for highly deacetylated and therefore nondegradable chitosan [51,52]. The incorporation of lysozyme into biodegradable CS(42)-TPP nanoparticles yielded a slower degradation of the nanoparticles compared to the addition of lysozyme to the preformed particles (particle size reduction of ≈30%, see Supplementary data, Figure S1). This indicates that the incorporation of lysozyme into the nanoparticles hinders the enzymatic degradation. Increasing the lysozyme concentration to 150 µg/mL clearly accelerates the degradation of CS(42)-TPP nanoparticles (particle size reduction of ≈60%, see Supplementary data, Figure S2). These results show that CS(42)-TPP nanoparticles can be degraded by lysozyme *in vitro* and confirm the strong influence of the degree of acetylation of the chitosan used.

### 2.5. Coated Titanium Substrates

Titanium and its alloys are commonly used materials for hip implants due to their good mechanical properties and biocompatibility. Thus, titanium substrates were utilized for the carried out coating experiments. The substrates were coated via spray-coating with pure CS(42)-TPP nanoparticle dispersions and drug loaded CS(42)-TPP nanoparticle dispersions. The coatings were characterized using ellipsometric measurements, which provide information about the layer thickness of the sprayed coatings, scanning electron microscopy (SEM) and X-ray photoelectron spectroscopy (XPS).

**Figure 3:**
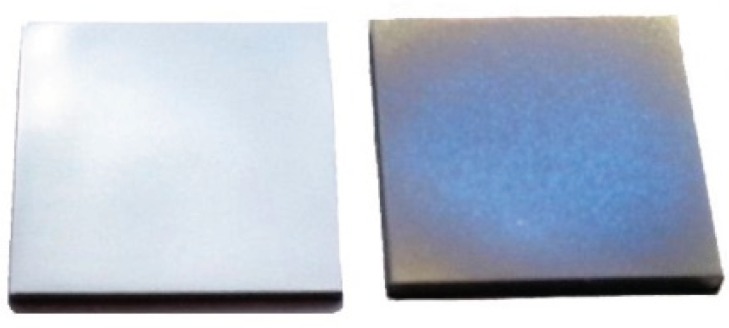
Uncoated, polished titanium substrate (**left**) and chitosan-tripolyphosphate spray-coated titanium substrate (**right**).

Titanium substrates were spray-coated for a defined period of time (3 min), see Figure 3. These coatings showed little deviation in regard to the resulting film thickness in the range of 76 ± 3 nm and were used for all further investigations.

**Figure 4:**
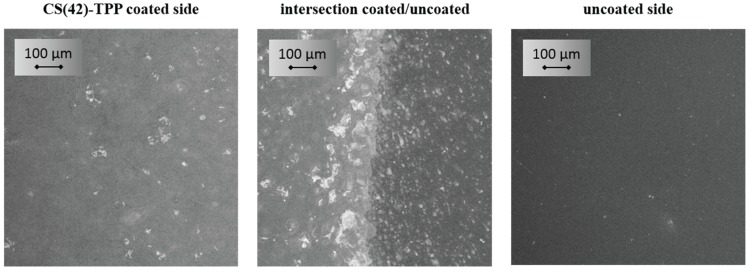
SEM pictures of spray-coated chitosan-tripolyphosphate nanoparticle solution films (3:1, 1 mg/mL, 0.075% AcOH, DA 42%) on a partially coated titanium substrate with a 250× magnification.

Comparing the resulting coating thickness with the hydrodynamic radius of the CS(42)-TPP nanoparticles it becomes obvious that the nanoparticles with about 150 nm are twice as big as the coating itself with ≈76 nm. Thus, we performed SEM measurements in order to validate whether there are nanoparticles on the titanium substrate or not. In Figure 4, showing a partially coated titanium substrate in a magnification of 250, a homogeneous film on the coated side and a clear intersection between the coated and the uncoated side is visualized. Raising the magnification to up to 200 k revealed that there were no nanoparticles on the coated sides of the titanium substrates. It seems that the nanoparticles are deformed during the spray-coating process and a homogeneous layer is formed. This behavior is not unexpected since the CS(42)-TPP nanoparticles possess a relatively low zeta potential of +23 mV which results in weak interparticular repulsion and low particle stability. Additionally, the phenomenon of aggregation or coalescence of solids induced by physical drying (evaporation of the solvent) is well known and the basic principle of water based varnish coatings [53].

**Figure 5:**
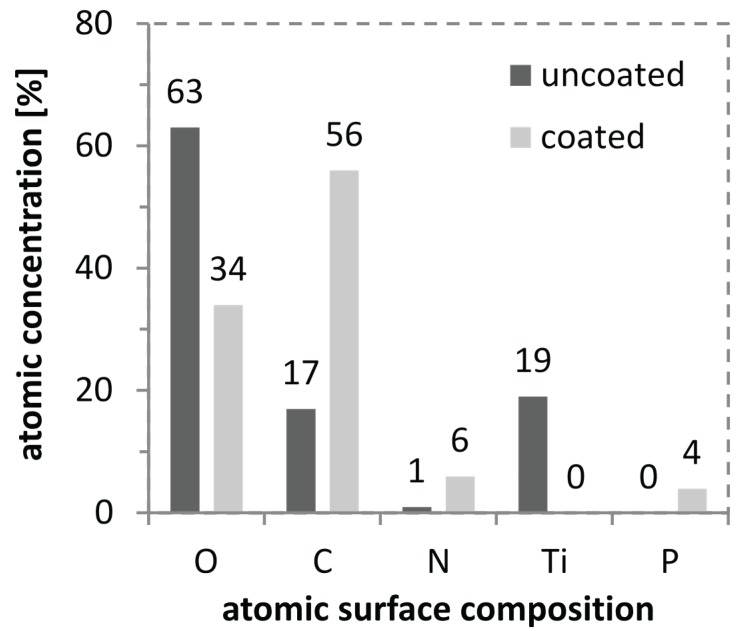
XPS measurement of a spray-coated chitosan-tripolyphosphate nanoparticle solution film (3:1, 1 mg/mL, 0.075% AcOH, DA 42%) on a titanium substrate and a blank substrate showing the atomic concentrations on the substrates surfaces.

For further characterization of the coated titanium substrates XPS measurements were carried out, which give insight into the elemental composition of a surface up to a depth of about 10 nm. As shown in Figure 5 for the uncoated surface oxygen and titanium are detected due to the passivizing titanium oxide layer. In addition signals for carbon and nitrogen are found, which are due to common impurities. In comparison, for the CS(42)-TPP nanoparticle coated surface no titanium signal can be detected which indicates a complete coverage of the surface with CS(42)-TPP. Additionally, a signal for phosphor appears and an increase in nitrogen and carbon is obvious. The phosphor can be assigned to the tripolyphosphate (TPP) while carbon, oxygen and nitrogen are present in chitosan. This proves the presence of chitosan and tripolyphosphate on the coated titanium substrates.

### 2.6. BMP-2 in CS-TPP Nanoparticles

Drug loading/release experiments were carried out using bone morphogenetic protein 2 (BMP‑2). Protein loading into CS-TPP nanoparticles can be achieved via two different methods which are reported in literature. One method is the incorporation of protein during the particle formation. The second method is based on the incubation of particles with a protein containing solution [28]. CS(42)-TPP nanoparticle drug loading properties were investigated using the incorporation method due to a higher loading efficiency compared to the incubation method as reported by Gan *et al.* [28].

CS(42)-TPP nanoparticles loaded with BMP-2 (51 µg/mL) have a greater particle size with a higher PDI compared to unloaded ones (146 ± 72 nm z-average unloaded to 227 ± 112 nm z-average loaded with BMP-2). The zeta potential also increases with the loading of BMP-2 into the particles from 23 ± 5 mV to 29 ± 5 mV for BMP-2 loaded nanoparticles. This observation meets our expectations due to the fact that BMP-2, with an isoelectric point of 8.5, is positively charged and should therefore increase the zeta potential of the particles upon incorporation [7,54]. For lower BMP‑2 concentrations the increase in size and zeta potential is less pronounced. The loading efficiency of BMP-2 into biodegradable CS(42)-TPP nanoparticles was quantified via ELISA and yielded incorporation values of 87% and higher. The release of BMP-2 from these nanoparticles could not be evaluated via ELISA due to the fact that the solubility of the particles changed as consequence of the acetylation procedure, resulting in particles that could not be separated from the mother solution via centrifugation.

### 2.7. Cell Response to BMP-2-Loaded Surfaces

**Figure 6:**
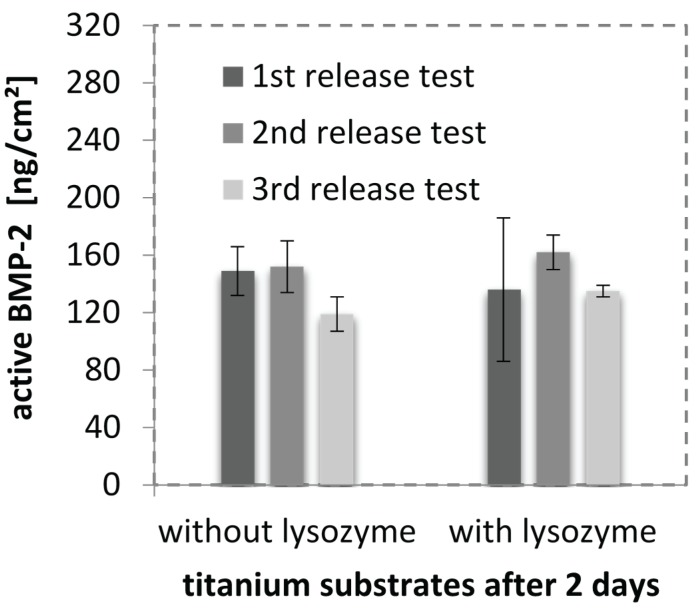
Active BMP-2 found on CS-TPP nanoparticle coated titanium substrates after 2 days quantified via BRE luciferase test. Shown are the results of three different experimental runs with triplicate samples tested with and without lysozyme for particle degradation.

Since we were able to show that BMP-2 is almost completely incorporated in CS(42)-TPP nanoparticles, spray-coating these particles and test the drug release from a coating seemed to be a promising approach for an *in vitro* drug release simulation. A test system based on genetically modified cells (BRE-Luc cells), which upon stimulation with BMP-2 produce luciferase, was employed in order to verify that there is biologically active BMP-2 released from or adhering to the coated titanium substrates. The results from the experiments carried out in triplicate are shown in Figure 6. The different experimental runs show good reproducibility. The volume coated per substrate was about 20 µL of the drug loaded nanoparticle dispersion (BMP-2: 16 µg/mL) resulting in an average BMP-2 load of 320 ng/cm^2^ (a preliminary conducted test series with Millipore revealed that in 3 min of spray-coating about 20 µL were used). The resulting coating thicknesses were comparable with pure nanoparticle coatings sprayed for 3 min. A BRE luciferase test revealed that BMP-2 could be detected as biologically active on the titanium substrates after 2 days. The detected activity is equivalent to a dose of 284 ng/mL BMP-2 in solution, which would equal 142 ng/cm^2^ on the titanium substrate. Interestingly, the addition of lysozyme had no influence on the amount of BMP-2 quantified. As mentioned above lysozyme is capable of degrading the nanoparticles and was thought to lead to an increased release of BMP-2 compared to the “without lysozyme” series. Taking into account that the release was conducted in cell medium (10% FBS) and that lysozyme is a serum protein, we concluded that there is lysozyme present in both series. Thus, the addition of lysozyme only raises the concentration slightly and does not seem to have a huge impact on the detected amount of BMP-2.

**Figure 7:**
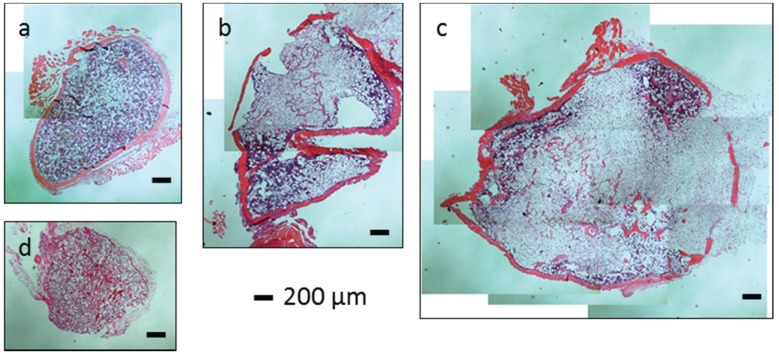
Microscopic analysis of ossicle sections after 4 weeks of implantation (H & E-staining). (**a**) implant: collagen sponge loaded with CS(42)-TPP nanoparticles containing 1 µg BMP-2; (**b**) implant: collagen sponge loaded with CS(42)-TPP nanoparticles containing 2 µg BMP-2; (**c**) implant: collagen sponge loaded with CS(42)-TPP nanoparticles containing 5 µg BMP-2 and (**d**) implant: collagen sponge loaded with CS(42)-TPP nanoparticles.

### 2.8. In Vivo Study: Implantation of Nanoparticles in Mice

In order to investigate whether the CS(42)-TPP nanoparticles negatively affects the action of BMP-2 *in vivo* or not, an animal study in mice was conducted. In the mouse with collagen sponges that contained pure BMP-2 only one ossicle could be found and explanted. This ossicle was unfortunately destroyed during the sectioning process, but its dimension was comparable to ossicles from BMP-2 loaded CS(42)-TPP nanoparticles (Figure 7). All mice implanted with BMP-2 loaded CS(42)-TPP nanoparticles developed ectopic bony elements. The dimension of an ossicle for 5 µg BMP-2 in CS-TPP nanoparticles was about 0.3 cm in diameter. In contrast, CS(42)-TPP nanoparticles without BMP-2 did not induce any bone formation. These results indicate that the bone-inducing capacity of BMP-2 is not negatively affected by the CS(42)-TPP nanoparticles. BMP-2 incorporated in nanoparticles is, therefore, accessible and/or released in a rate comparable with pure BMP-2 and able to induce bone formation *in vivo* in mice.

## 3. Experimental Section

### 3.1. Materials

Chitosan (medium molecular weight, 190–310 kDa, degree of acetylation (DA) 15%–25%) was purchased from Sigma-Aldrich (St. Louis, MO, USA) and purified as described below. Pentasodium tripolyphosphate (98%) was also bought from Sigma-Aldrich and used as delivered. Acetic acid 100% from Sigma-Aldrich was filtered through a 0.22 µm Millipore Millex-GP PES filter before use. Acetic anhydride (99%) was purchased from Sigma-Aldrich and used as delivered. Phosphate buffered saline tablets (pH 7.4) were purchased from Sigma and used according to the instructions. Recombinant human BMP‑2 was prepared from *E. coli* as described in literature and provided by the Center for Infection Research Braunschweig [55]. Chicken egg white lysozyme (hydrochloride form) was purchased from Serva (Heidelberg, Germany). Titanium substrates (1 mm thick, Goodfellow) consisting of Ti6Al4V were cut into 1 × 1 cm^2^ squares and treated as described below. Acetone, methanol, ethanol and dichloromethane were distilled before use.

### 3.2. Chitosan Purification

Purification of chitosan has been accomplished according to a method reported by Gan *et al.* [37]. Briefly, chitosan was dispersed in 1 M sodium hydroxide (1 g CS/10 mL NaOH) and heated to 70 °C for 2 h. Subsequently the chitosan was filtered off, washed with deionized water and dissolved in 1% (w/v) acetic acid. The chitosan solution was then filtered, dialyzed (10 kDa·MW cutoff) in deionized water and finally lyophilized.

### 3.3. Chitosan Acetylation

Chitosan acetylation was carried out following a method published by Freier *et al.* [38]. Briefly, purified chitosan with a DA of 17% was dissolved in 1% (v/v) acetic acid to yield a 5 mg/mL acetic chitosan solution. Afterwards the solution volume was doubled with distilled ethanol. Then an amount of acetic anhydride calculated according to Freier *et al.* was added and the mixture was stirred for 18 h. The acetylated chitosan with a DA of 42% was then purified via dialysis, subsequently lyophilized and characterized by ^1^H-NMR.

### 3.4. NMR Characterization

NMR spectra were acquired employing the spectrometer DRX 400 (Bruker, Billerica, MA, USA) with a magnetic field strength of 9.4 T. D_2_O, DCl and CD_3_COOD were used as deuterated solvents.

### 3.5. Viscometry

A viscosity measuring unit AVS 470 from Schott Instruments (Mainz, Germany) in combination with a transparent thermostat CT 72/P from SI Analytics was employed for viscosity measurements with a DIN IIc Ubbelohde capillary at 37 °C. For the experiments a 10 mg/mL CS(42) solution in 0.1% AcOH was prepared and tempered at 37 °C. Afterwards, 1.5 µg lysozyme per milliliter chitosan solution were added and the execution time was measured for 24 days. 0.1% AcOH was used as reference. The kinematic viscosity was calculated as follows (Equation (1)):

(1) ν=K×t

with *K* = 0.3 (instrument constant) and *t* = measured execution time.

### 3.6. Nanoparticle Synthesis

CS(42)-TPP nanoparticles were prepared according to the following procedure: Purified, acetylated chitosan was dissolved in 0.1% (v/v) acetic acid to yield a 1 mg/mL CS solution. After complete dissolution a 1 mg/mL TPP in Millipore solution was flush mixed with the CS solution. Analysis of particles’ size and zeta potential was performed right after the synthesis of the particles.

### 3.7. Particle Size and Zeta Potential

Particle size and zeta potential measurements were carried out using a Zetasizer Nano ZS from Malvern Instruments (Malvern, UK). For size measurements disposable sizing cuvettes (DTS0012) and for zeta potential measurements clear disposable zeta cells (DTS1060C) were used. All measurements were conducted at 20 °C except the particle degradation experiments for which a temperature of 37 °C was set. Malvern Zetasizer Software Version 6.32 was used for data evaluation.

### 3.8. Nanoparticle Degradation

The degradation of CS(42)-TPP nanoparticles was carried out by adding lysozyme to the nanoparticle dispersion. 990 µL CS(42)-TPP nanoparticle dispersion (1 mg/mL) were filled in a Zetasizer sizing cell and maintained at 37 °C. Subsequently lysozyme was added to the solution in order to reach a concentration of 1.5 µg/mL or 150 µg/mL. In case of nanoparticles being loaded with lysozyme the enzyme was premixed with the chitosan solution and after that cross linked with TPP. The ongoing degradation process was monitored via continuous size measurements using the Zetasizer Nano ZS.

### 3.9. Surface Modification

The modification of Ti6Al4V substrates surfaces with CS-TPP nanoparticles was carried out using the following procedure: The substrates were grinded, polished and cleaned by ultrasonification in distilled dichloromethane, acetone, methanol and Millipore water. The washed substrates were then plasma cleansed and used for surface modification experiments. Coating of the titanium substrates was performed by manual spray-coating with an airbrush Aztek A470 from Testors (Vernon Hills, IL, USA). CS(42)-TPP nanoparticles were sprayed onto the titanium substrates for a specific time, e.g., 3 min. Subsequently the platelets were shaken in Millipore water and dried.

### 3.10. Ellipsometry

For ellipsometric measurements a Multiskop from Optrel (Sinzing, Germany) was used in ellipsometry mode. Each titanium plate was first measured as uncoated sample and used as reference for the corresponding coated sample. Measurements were carried out using the x,y-mode at 70°, scanning the substrate’s surface for a total of 16 data points. Data evaluation was carried out using Elli Version 3.2 from Optrel.

### 3.11. Scanning Electron Microscopy

SEM measurements were carried out using the LEO 1550 made by Carl Zeiss (Oberkochen, Germany). Coated titanium substrates, as described above, were investigated.

### 3.12. X-ray Photoelectron Spectroscopy

XPS measurements were conducted using a PHI 6500 ci from Physical Electronics (Ismaning, Germany) equipped with a monochromatized Al Kα X-ray source. All spectra were taken at a 0° take-off-angle. Data analysis was performed with the CasaXPS software from Casa Software Ltd. (Teignmouth, UK).

### 3.13. Cell Response to BMP-2-Loaded Surfaces

To determine the BMP-2 release from coatings, BMP-2 was first incorporated in CS-TPP nano-particles. In brief, BMP-2 was added to a 0.1% acetic acid solution containing CS(42) at 1 mg/mL. After vortexing the solution, the TPP solution was injected. The mixing ratio of CS-TPP nanoparticles was 3:1 containing 16 µg BMP-2 per milliliter. The prepared particles were vortexed and 15 min later titanium substrates were coated. Drug loaded nanoparticles were sprayed for 3 min onto titanium platelets as described above. The sterilization of the coated substrates was carried out by UV treatment of both sides for 20 min. Then, each of the substrates was placed in a well and washed with 1 mL PBS which was drawn off after brief shaking. Subsequently, the bioactive BMP-2 in the nanoparticles on the platelets was quantified by the BRE-Luc-test as described by Ehlert *et al.* [56]. In short, C2C12/BRE-Luc cells were seeded in 1 mL at 35,000 c/well of 24-well plates. The cells were allowed to adhere to the nanoparticle-coated titanium platelets for 2 h before the excessive solution was removed. Finally, 500 µL of 10% FCS or 495 µL of 10% FCS plus 5 µL lysozyme (150 µg/mL in Millipore water) were added to each hole of the well plates. The well plates were stored at 37 °C at 98% humidity in an incubator for 2 days and then taken out for analysis. The wells were washed with PBS and then frozen at −70 °C for a minimum of 1 h. Cell lysis was conducted using CAT lysis buffer (from CAT ELISA kit, Roche, Mannheim, Germany) with protease inhibitors (Roche, Mannheim, Germany) (70 µL per substrate). The cells were scraped off the substrates, transferred into Eppendorf tubes and centrifuged at 13 k·rpm, 4 °C for 10 min. Luciferase testing was done in white 96 well plates using 5 µL supernatant from the centrifugation with 25 µL luciferin solution. The amount of BMP-2 released from coating was calculated from the standards by linear or polynominal regression analysis after correction for background (photon counts at 0 ng BMP-2). Luciferase is only transcribed when the BMP-dependent Smad-signaling cascade is intracellularly activated *i.e.*, this assay quantifies biologically active BMP-2.

### 3.14. In Vivo Study: Implantation of Nanoparticles in Mice

BMP-2 loaded CS(42)-TPP nanoparticles (3:1, 1 mg/mL, 0.075% AcOH) were prepared as described above with 100 µg/mL, 200 µg/mL and 500 µg/mL BMP-2. 10 µL of the BMP2- loaded nanoparticles in 3 × 3 mm collagen sponges were implanted into the right and left caudal thigh muscles of three female C3H/HeNHSd mice (Harlan; 6–7 weeks old), one mouse for each BMP-2 concentration. As a control, CS(42)-TPP nanoparticles without BMP-2 or pure BMP-2 in collagen sponges were implanted as well. Four weeks later the implants were explanted and stored overnight (4 °C) in 1 mL paraformaldehyde (4% in PBS). Then, implants were decalcified in 1 mL 16.8% EDTA in PBS at room temperature (one week). The implants were embedded in paraffin and sectioned with a microtome. Subsequently, sections on glass slides were subjected to H&E staining and analyzed with a light microscopy.

## 4. Conclusions

Biodegradable chitosan (CS)-tripolyphosphate (TPP) nanoparticles (148 nm z-Average) could be obtained using chitosan with a degree of acetylation (DA) of 42%. These biodegradable particles needed to be developed because preliminary experiments revealed that the release of bone morphogenetic protein 2 (BMP-2) from CS-TPP nanoparticles is not based on diffusion. The CS (DA 42%) particles proved to possess a dramatically increased biodegradation rate using lysozyme (40% decrease in diameter after 4 days) compared to CS(17)-TPP nanoparticles (no effect after 7 days). The degradation of CS(42) was additionally confirmed via viscometry. Upon incorporation of BMP-2 into the nanoparticles an increase in size and zeta potential could be observed. ELISA quantification stated a drug loading efficiency of >87%. Spray-coating of titanium substrates with biodegradable CS(42)-TPP nanoparticles led to the formation of visible films with thicknesses of ≈80 nm and good reproducibility. SEM and XPS measurements confirmed the nanoparticle coating on the spray-coated titanium substrates and indicate that the nanoparticles reorganize into a homogeneous film on the titanium substrates. The examination of such films in our BRE luciferase studies shows that we were able to create biodegradable nanofilm coated titanium surfaces loaded with biologically active BMP-2. The biologic activity of the incorporated BMP-2 was as well observed in an *in vivo* study in mice. Here, a dose dependent induction of ectopic bone growth seems to occur for BMP-2 loaded CS(42)-TPP nanoparticles.

This article indicates the potential of biodegradable CS(42)-TPP nanoparticles for a fast release of unmodified BMP-2 at the implant surface in combination with a high release efficiency on account of the biodegradability of the system.

## Supplementary Files

Supplementary File 1
