# Supplementary material for: Biodegradable Chitosan Nanoparticle Coatings on Titanium for the Delivery of BMP-2
Source: Biomolecules. 2015 Jan 8;5(1):3–19. doi: 10.3390/biom5010003 (PMC4384108; doi:10.3390/biom5010003)
Supplement: Supplementary File 1 [file biomolecules-05-00003-s001.pdf]

Supplementary Files

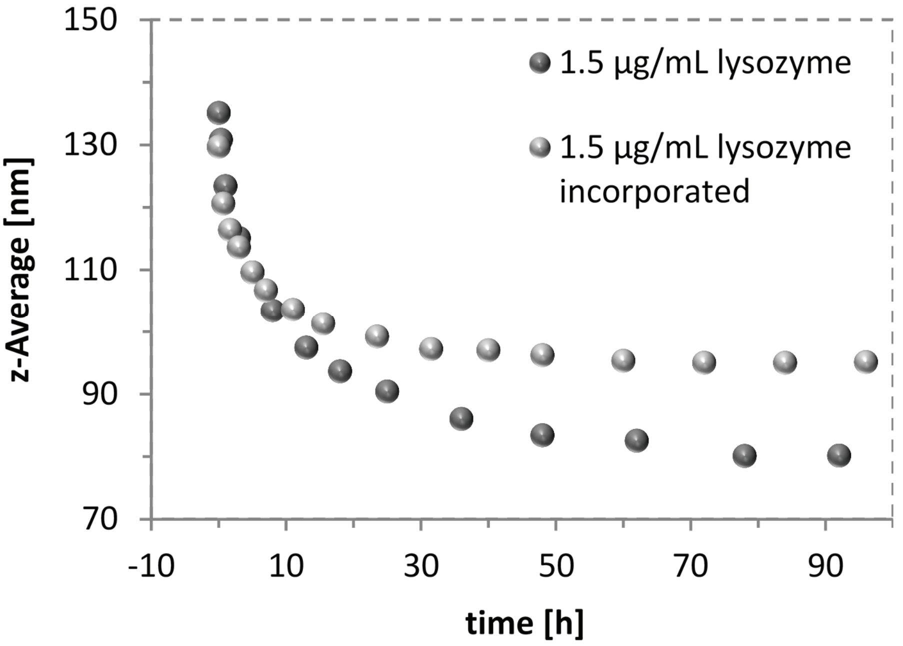

Figure S1. Plot of the particle size over the time during the degradation with 1.5 µg/mL lysozyme (“incorporated” and “free”) at 37 °C for chitosan-tripolyphosphate (3:1, 1 mg/mL, 0.075% AcOH, DA 42%) nanoparticle solutions.

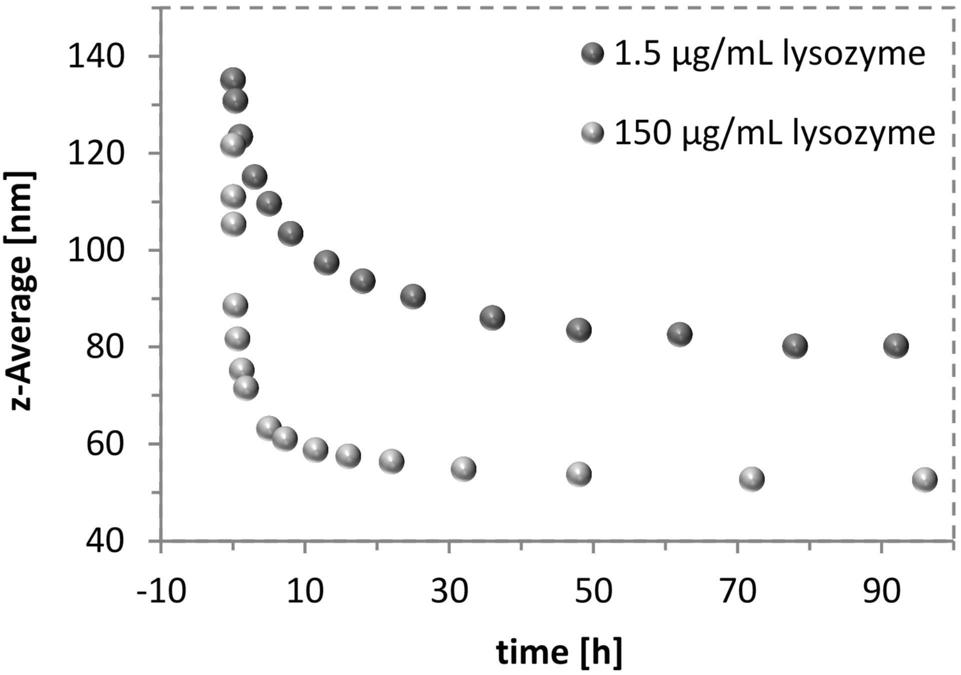

Figure S2. Plot of the particle size over the time during the degradation with 1.5 µg/mL or 150 µg/mL lysozyme at 37 °C for chitosan-tripolyphosphate (3:1, 1 mg/mL, 0.075% AcOH, DA 42%) nanoparticle solutions.

© 2015 by the authors; licensee MDPI, Basel, Switzerland. This article is an open access article distributed under the terms and conditions of the Creative Commons Attribution license (http://creativecommons.org/licenses/by/4.0/).
